# Heart Rate and Respiratory Rate Influence on Heart Rate Variability Repeatability: Effects of the Correction for the Prevailing Heart Rate

**DOI:** 10.3389/fphys.2016.00356

**Published:** 2016-08-18

**Authors:** Jakub S. Gąsior, Jerzy Sacha, Piotr J. Jeleń, Jakub Zieliński, Jacek Przybylski

**Affiliations:** ^1^Cardiology Clinic of Physiotherapy Division of the 2nd Faculty of Medicine, Medical University of WarsawWarsaw, Poland; ^2^Faculty of Physical Education and Physiotherapy, Opole University of TechnologyOpole, Poland; ^3^Department of Biophysics and Human Physiology, Medical University of WarsawWarsaw, Poland; ^4^Interdisciplinary Centre for Mathematical and Computational Modelling, University of WarsawWarsaw, Poland

**Keywords:** heart rate, heart rate variability, heart rate correction, respiratory rate, repeatability, autonomic nervous system, autonomic cardiac control

## Abstract

**Background:** Since heart rate variability (HRV) is associated with average heart rate (HR) and respiratory rate (RespRate), alterations in these parameters may impose changes in HRV. Hence the repeatability of HRV measurements may be affected by differences in HR and RespRate. The study aimed to evaluate HRV repeatability and its association with changes in HR and RespRate.

**Methods:** Forty healthy volunteers underwent two ECG examinations 7 days apart. Standard HRV indices were calculated from 5-min ECG recordings. The ECG-derived respiration signal was estimated to assess RespRate. To investigate HR impact on HRV, HRV parameters were corrected for prevailing HR.

**Results:** Differences in HRV parameters between the measurements were associated with the changes in HR and RespRate. However, in multiple regression analysis only HR alteration proved to be independent determinant of the HRV differences—every change in HR by 1 bpm changed HRV values by 16.5% on average. After overall removal of HR impact on HRV, coefficients of variation of the HRV parameters significantly dropped on average by 26.8% (*p* < 0.001), i.e., by the same extent HRV reproducibility improved. Additionally, the HRV correction for HR decreased association between RespRate and HRV.

**Conclusions:** In stable conditions, HR but not RespRate is the most powerful factor determining HRV reproducibility and even a minimal change of HR may considerably alter HRV. However, the removal of HR impact may significantly improve HRV repeatability. The association between HRV and RespRate seems to be, at least in part, HR dependent.

## Introduction

The analysis of heart rate variability (HRV) has been widely used to non-invasively investigate the cardiac autonomic regulation in healthy subjects and patients with various diseases (Billman, 2011). Decreased HRV indicates the imbalance of the autonomic nervous control of heart rate and may predict adverse outcomes including all-cause mortality (Dekker et al., 2000). On the other hand, high HRV is associated with a good prognosis in both healthy and disease states (Zulfiqar et al., 2010).

From the point of view of medical practice, it is important to evaluate physiological and pathological phenomena using reliable and validated tools to ensure reproducible results and present meaningful findings (Lachin, 2004). There exist only few studies on the reproducibility of HRV indices calculated on the basis of short-term (5–7 min) stable ECG recordings in healthy young adults (Sinnreich et al., 1998; Jáuregui-Renaud et al., 2001; Carrasco et al., 2003; Sandercock et al., 2005; McNames and Aboy, 2006; Guijt et al., 2007; Pinna et al., 2007; Tannus et al., 2013). However, the authors of these studies did not consider an interaction between HRV and average heart rate (HR; Sacha, 2013, 2014a,b,c; Sacha et al., 2013a,b,c, 2014; Monfredi et al., 2014; Stauss, 2014; Billman et al., 2015) and its influence on the reproducibility of HRV analysis (Sacha et al., 2013c). Since HRV is primarily HR dependent, different HR may exert various impact on HRV values (Sacha and Grzeszczak, 2001; Sacha and Pluta, 2005a,b, 2008; Sacha, 2013, 2014a,b,c; Sacha et al., 2013a,b,c, 2014; Monfredi et al., 2014; Stauss, 2014; Billman et al., 2015; Gąsior et al., 2015). Therefore, it was suggested that an adequate correction designed to remove the HR influence on HRV should be performed before drawing ultimate conclusions about HRV corresponding to different HR (Sacha and Pluta, 2008; Sacha, 2013, 2014a; Sacha et al., 2013a; Monfredi et al., 2014; Stauss, 2014; Billman et al., 2015). Recently, Sacha et al. noticed that HR is a powerful factor of the HRV reproducibility, i.e., HRV corrected for HR turned out to be significantly more reproducible than the standard one (Sacha et al., 2013c). The observation was made among healthy participants who recorded their heart rhythm twice daily over 30 days (Sacha et al., 2013c).

Another relevant factor influencing HRV is breathing, particularly, the respiratory frequency may considerably modify HRV (Bruce, 1996; Billman, 2011; Quintana and Heathers, 2014; Quintana et al., 2016). In fact, majority of the studies addressing the short-term HRV did not examine changes in respiratory rate between HRV measurements as a potential factor disturbing their reproducibility.

In the present study we investigated whether the impact of HR and respiratory rate on the HRV repeatability could be detectable even in two separate measurements performed in stable and comparable circumstances. To this end, we evaluated differences between two short-term HRV measurements among healthy adults and their association with differences in HR and respiratory rate. We also checked whether the exclusion of HR impact on HRV might improve the agreement between the HRV measurements.

## Materials and methods

### Participants

Forty students voluntarily took part in the study. Before the experiment all participants filled in a questionnaire regarding chronic diseases. None of them was taking any medication and had any history of chronic illnesses. Participants were carefully instructed to abstain from alcohol, caffeine, smoking and intensive physical efforts starting from the afternoon of the day before the ECG examinations and have usual meals on the study days. They all had received information about the study and gave their informed written consent. The research was approved by the University Bioethical Committee and followed the rules and principles of the Helsinki Declaration.

### ECG acquisition

Each participant underwent two ECG examinations 7 days apart—the first examination was denoted as “Test” while the second one as “Retest.” Both examinations (i.e., Test and Retest) were performed under the same conditions—in a quiet, bright university room, with stable temperature and humidity. Twelve-lead, 5-min ECG recordings were performed in a supine position at about 12:00 pm before lunch. All ECG signals were recorded with sampling frequency of 500 Hz and stored on a computer hard disc using Cardiv—cardiovascular system software (Institute of Medical Technology and Equipment, Zabrze, Poland). On both study days, in order to stabilize HR, the participants were asked to lie in supine position about 5 min and then the appropriate ECG recordings started. They were also encouraged to breathe naturally and refrain from speaking and moving during the ECG examination.

### ECG derived respiration

The ECG derived method to find a rate of respiration was used according to Sinnecker et al. (2014). The respiratory rate was estimated from the main modulation of QRS amplitude which is supposed to be caused by breathing. For each of the 12 ECG leads the time series of QRS amplitude was computed and then local maxima (i.e., data points with values greater than both the preceding and the following data point) were identified. Subsequently, the mean interval between consecutive local maxima for each time series was calculated and the reciprocal value of the mean maximum-to-maximum interval was obtained. The respiratory rate was calculated as the median of these reciprocal values over all time series (Sinnecker et al., 2014).

### HRV analysis

Prior to HRV analysis the ECG recordings were visually inspected for potential non-sinus or aberrant beats. The erroneous beats were manually corrected, i.e., one R-R interval before and after each non-sinus beat were eliminated and replaced by R-R intervals computed by interpolation of degree zero based on the surrounding normal beats (Peltola, 2012). HRV analysis was performed on 5-min ECG time series by using Kubios HRV 2.1 software (University of Eastern Finland, Kuopio, Finland; Tarvainen et al., 2014). Time and frequency domain measures of HRV were calculated according to Task Force of the European Society of Cardiology and the North American Society of Pacing and Electrophysiology guidelines (Task Force of the European Society of Cardiology and the North American Society of Pacing and Electrophysiology, 1996). The following time-domain parameters were determined: standard deviation of R-R intervals (SDNN), root mean square of successive R-R interval differences (RMSSD) and pNN50 which denotes percent of R-R intervals differing >50 ms from the preceding one (Task Force of the European Society of Cardiology and the North American Society of Pacing and Electrophysiology, 1996).

Before calculating spectral HRV parameters, the smoothness priors based detrending approach was employed (smoothing parameter, Lambda value = 1000; Tarvainen et al., 2002). The R-R interval series were transformed to evenly sampled time series with 4-Hz resampling rate. The detrended and interpolated R-R interval series were used for the frequency-domain HRV analysis. HRV spectra were calculated by using the fast-Fourier-transform (FFT) with Welch's periodogram method (50% overlap window and 60 s window width). The following spectral components were distinguished: low frequency (LF, 0.04–0.15 Hz), high frequency (HF, 0.15–0.40 Hz), and total power (TP, 0–0.4 Hz) in absolute units (ms^2^), and nLF, nHF in normalized units (nu), as well as LF/HF ratio according to the guidelines (Task Force of the European Society of Cardiology and the North American Society of Pacing and Electrophysiology, 1996).

### HRV correction

To investigate the impact of HR on HRV, standard HRV parameters (i.e., those in absolute units, ms^2^) were corrected with respect to an average HR. If HRV parameters revealed a negative correlation with HR, they were divided by suitable powers of their corresponding average R-R intervals, however, if they presented a positive correlation, the correction relied on multiplication by adequate powers of average R-R intervals (Sacha et al., 2013a,c).

### Statistical analysis

The Kolmogorov-Smirnov test was used to assess the normality of the data distribution. Wilcoxon signed-rank test or Student's paired *t*-test was employed to compare systematic changes between Test and Retest in analyzed parameters. Spearman's rank correlation coefficient (R) or Pearson's correlation coefficient (r) were used to assess the relationship between variables. Multiple regression analysis was carried out to identify independent determinants of differences between Test and Retest in standard and corrected HRV. The regression equation was used to compute percentage changes in HRV per 1 bpm change in HR between the examinations. Bland-Altman plots were produced to allow visualization of any systematic change between the Test and Retest in analyzed HRV parameters (Bland and Altman, 1986, 1999). The within-subject coefficient of variation (CV) was calculated to assess repeatability. The threshold probability of *p* < 0.05 was taken as the level of significance for all statistical tests. All calculations were performed using the STATISTICA 12-StatSoft. Inc software (Tulsa, USA). The Bland-Altman plots were created using Graph Pad Prism 5 (Graph Pad Software Inc., San Diego, CA, USA, 2005).

## Results

Four participants out of 40 were excluded from the analysis due to incomplete ECG data. Consequently, 36 (22 males) young healthy adults (mean age: 22.5 years, SD: 1.9, range: 18–26 years) took part in the study. There was no consistent difference between Test and Retest in HR (74.7 ± 11.9 vs. 73.6 ± 11.8, *p* = 0.49), RespRate (17.2 ± 3.4 vs. 17.0 ± 3.2, *p* = 0.52) and any standard HRV parameter (*p* ≥ 0.29 for all).

The following HRV indices: SDNN, RMSSD, pNN50, LF, HF, nHF, and TP were negatively correlated with HR and RespRate with R ranging between: −0.40 to −0.84 (*p* < 0.05 for all) and −0.35 to −0.66 (*p* < 0.05 for all), respectively. The nLF and LF/HF positively correlated with HR and RespRate with R ranging between: 0.40–0.55 (*p* < 0.05 for both) and 0.35–0.36 (*p* < 0.05 for both), respectively.

There was a significant positive correlation between HR and RespRate in Test (*r* = 0.36, *p* < 0.05) and Retest (*r* = 0.44, *p* < 0.01), moreover, the Test–Retest difference in HR (HR-diff) correlated with the Test–Retest difference in RespRate (RespRate-diff; *r* = 0.57; *p* < 0.001). The differences between Test and Retest of most HRV parameters were significantly related with HR-diff and RespRate-diff (Table 1). However, in the multiple regression analysis, only HR-diff proved to be an independent determinant for all time domain HRV indices and TP—in the case of LF and HF this determination was statistically borderline (Table 2). Indeed, RespRate-diff seemed to be redundant in these regression models since it was more tightly associated with HR-diff (*r* = 0.57) than with HRV parameters (Table 1; Kraha et al., 2012). The additional regression analysis (without RespRate-diff as an independent variable) showed that HR-diff was the only significant determinant for all time and frequency domain (i.e., those in absolute units, ms^2^) HRV parameters with β-values ranging between: −0.48 to −0.67 (*p* < 0.01 for all). Importantly, every change in HR by 1 bpm between the two examinations changed the HRV values by the following percent: 4% (SDNN), 6% (RMSSD), 56% (pNN50), 8% (LF), 15% (HF), 10% (TP)—i.e., by 16.5% on average. In the case of nLF, nHF and LF/HF, the regression models (both with and without RespRate-diff as an independent determinant) turned out to be not statistically significant (Table 2).

**Table 1:** **Correlations of Test–Retest differences in HRV parameters with Test–Retest differences in HR (HR-diff) and RespRate (RespRate-diff)**.

**Test–Retest Difference**	**HR-diff**		**RespRate-diff**	
	***r***	***p***	***r***	***p***
SDNN (ms)	−0.66	< 0.001	−0.50	< 0.01
RMSSD (ms)	−0.67	< 0.001	−0.41	< 0.05
pNN50 (%)	−0.64	< 0.001	−0.31	0.07
LF (ms2)	−0.49	< 0.01	−0.45	< 0.01
HF (ms2)	−0.52	< 0.01	−0.50	< 0.01
TP (ms2)	−0.58	< 0.001	−0.54	< 0.01
nLF (nu)	0.34	< 0.05	0.21	0.21
nHF (nu)	−0.34	< 0.05	−0.21	0.21
LF/HF	0.21	0.22	0.18	0.28

**Table 2:** **Results of the multiple regression analysis considering differences in HR (HR-diff) and RespRate (RespRate-diff), sex, and age as determinants of differences between Test and Retest in standard HRV parameters**.

**Test–Retest difference**	**Parameters of multiple regression analysis**					
	**Determinant**	**β**	***p***	**Multiple *R*^2^**	***F*-test**	***p***
SDNN (ms)	HR-diff	−0.57	< 0.01	0.46	6.7	< 0.001
	RespRate-diff	−0.19	0.26			
	Sex	0.02	0.90			
	Age	−0.05	0.70			
RMSSD (ms)	HR-diff	−0.64	< 0.001	0.45	6.3	< 0.001
	RespRate-diff	−0.03	0.85			
	Sex	−0.06	0.69			
	Age	−0.04	0.77			
pNN50 (%)	HR-diff	−0.64	< 0.001	0.41	5.4	< 0.01
	RespRate-diff	0.09	0.61			
	Sex	−0.01	0.93			
	Age	−0.01	0.97			
LF (ms^2^)	HR-diff	−0.35	0.07	0.29	3.1	< 0.05
	RespRate-diff	−0.25	0.19			
	Sex	−0.02	0.90			
	Age	−0.09	0.56			
HF (ms^2^)	HR-diff	−0.35	0.06	0.34	4.1	< 0.01
	RespRate-diff	−0.32	0.09			
	Sex	−0.01	0.97			
	Age	−0.11	0.48			
TP (ms^2^)	HR-diff	−0.41	< 0.05	0.41	5.4	< 0.01
	RespRate-diff	−0.32	0.08			
	Sex	−0.01	0.96			
	Age	−0.12	0.41			
nLF (nu)	HR-diff	0.31	0.14	0.14	1.3	0.31
	RespRate-diff	0.02	0.94			
	Sex	0.001	0.99			
	Age	−0.15	0.37			
nHF (nu)	HR-diff	−0.31	0.14	0.14	1.3	0.31
	RespRate-diff	−0.02	0.94			
	Sex	0.001	0.99			
	Age	0.15	0.37			
LF/HF	HR-diff	0.14	0.50	0.11	1.0	0.43
	RespRate-diff	0.11	0.62			
	Sex	−0.11	0.55			
	Age	−0.23	0.18			

*All independent variables (Test-Retest differences in HRV parameters) and determinants (HR-diff and RespRate-diff) presented normal distribution*.

To exclude the overall HR impact on HRV, the standard HRV parameters were corrected for their prevailing HR. HRV lost their dependence on HR after dividing SDNN, RMSSD, pNN50, LF, HF, TP and nHF by average R-R intervals to the power: 2, 3, 7, 2, 4, 3, and 1, respectively, however nLF and LF/HF stopped being dependent on HR after multiplying by average R-R intervals to the power 1 and 2, respectively. The same powers of average R-R intervals were used for the correction in Test and Retest.

There was no significant difference in the corrected HRV parameters between the 2 study days (*p* ≥ 0.15 for all). After correction (i.e., after overall exclusion of HR impact on HRV), the coefficients of correlation between RespRate and HRV decreased for all parameters, i.e., from −0.53, *p* < 0.0001 to −0.34, *p* < 0.01 (SDNN); from −0.49, *p* < 0.0001 to −0.25, *p* < 0.05 (RMSSD); from −0.49, *p* < 0.0001 to −0.3, *p* < 0.05 (pNN50); from −0.48, *p* < 0.0001 to −0.42, *p* < 0.001 (LF); from −0.64, *p* < 0.0001 to −0.54, *p* < 0.0001 (HF); from −0.59, *p* < 0.0001 to −0.49, *p* < 0.0001 (TP); from 0.35, *p* < 0.01 to 0.16, *p* = 0.19 (nLF); from −0.35, *p* < 0.01−0.17, *p* = 0.16 (nHF); and from 0.35, *p* < 0.01 to 0.17, *p* = 0.15 (LF/HF).

To find independent determinants of the differences in corrected HRV indices between Test and Retest, the multiple regression analysis was performed with RespRate-diff, sex and age as potential determinants. No regression model provided a significant multiple *R*^2^-value, although RespRate-diff was significantly associated with differences in corr-HF and corr-TP (Table 3).

**Table 3:** **Results of the multiple regression analysis considering differences in respiratory rate (RespRate-diff), sex and age as determinants of differences between Test and Retest in corrected HRV parameters**.

**Test–Retest Difference**	**Parameters of multiple regression analysis**					
	**Determinant**	**β**	***p***	**Multiple *R*^2^**	***F*-test**	***p***
corr-SDNN	RespRate-diff	−0.32	0.08	0.09	1.1	0.36
	Sex	0.13	0.48			
	Age	0.02	0.93			
corr-RMSSD	RespRate-diff	−0.17	0.36	0.05	0.5	0.67
	Sex	0.07	0.72			
	Age	0.13	0.47			
corr-pNN50	RespRate-diff	−0.16	0.37	0.10	1.2	0.35
	Sex	0.21	0.25			
	Age	0.22	0.21			
corr-LF	RespRate-diff	−0.32	0.08	0.09	1.1	0.36
	Sex	0.06	0.74			
	Age	−0.02	0.89			
corr-HF	RespRate-diff	−0.42	< 0.05	0.17	2.2	0.11
	Sex	0.15	0.37			
	Age	0.07	0.65			
corr-TP	RespRate-diff	−0.42	< 0.05	0.16	2.0	0.13
	Sex	0.14	0.41			
	Age	0.01	0.95			
corr-nLF	RespRate-diff	−0.09	0.60	0.10	1.1	0.35
	Sex	0.21	0.24			
	Age	−0.23	0.19			
corr-nHF	RespRate-diff	0.09	0.60	0.10	1.1	0.35
	Sex	−0.21	0.24			
	Age	0.23	0.19			
corr-LF/HF	RespRate-diff	0.07	0.72	0.06	0.7	0.58
	Sex	−0.13	0.49			
	Age	−0.21	0.23			

*All independent variables (Test-Retest differences in corrected HRV parameters) and determinant (RespRate-diff) presented normal distribution*.

**Figure 1:**
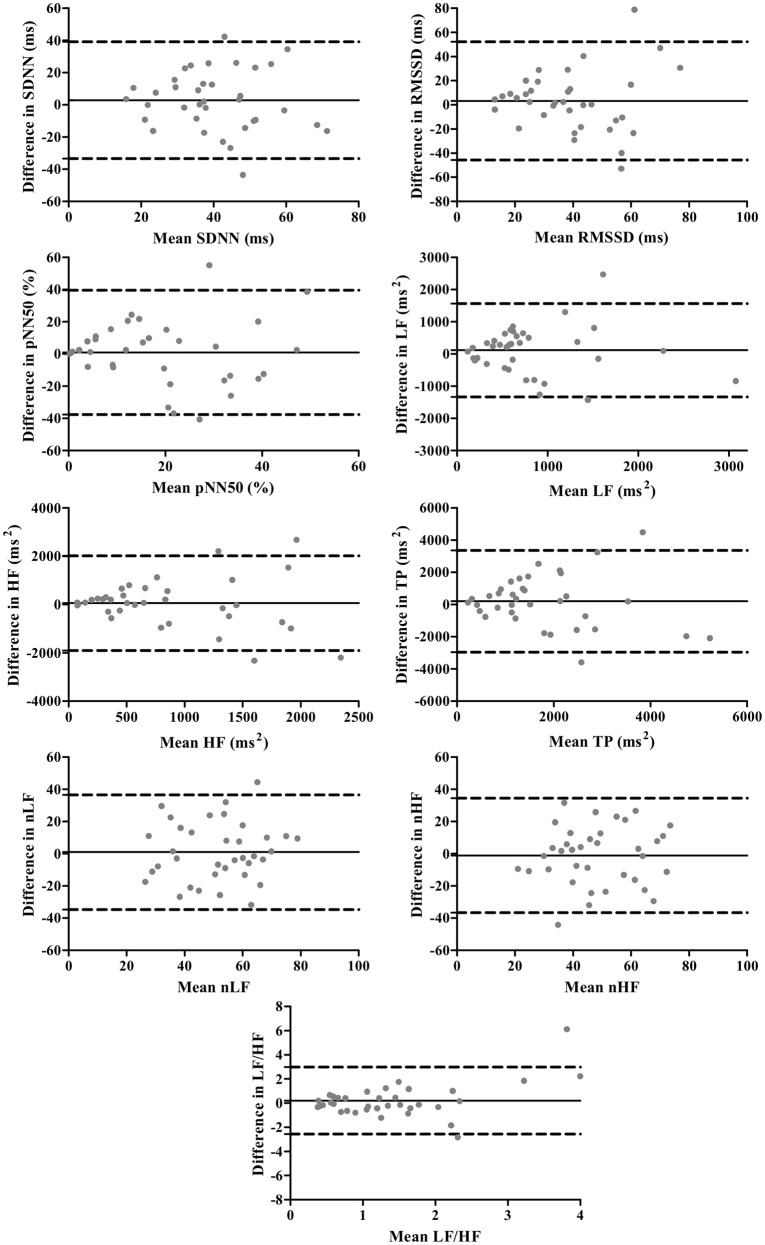
**Bland-Altman plots for standard HRV parameters**. The solid lines indicate the bias, and dotted lines are the 95% limits of agreement (±1.96 *SD*).

**Figure 2:**
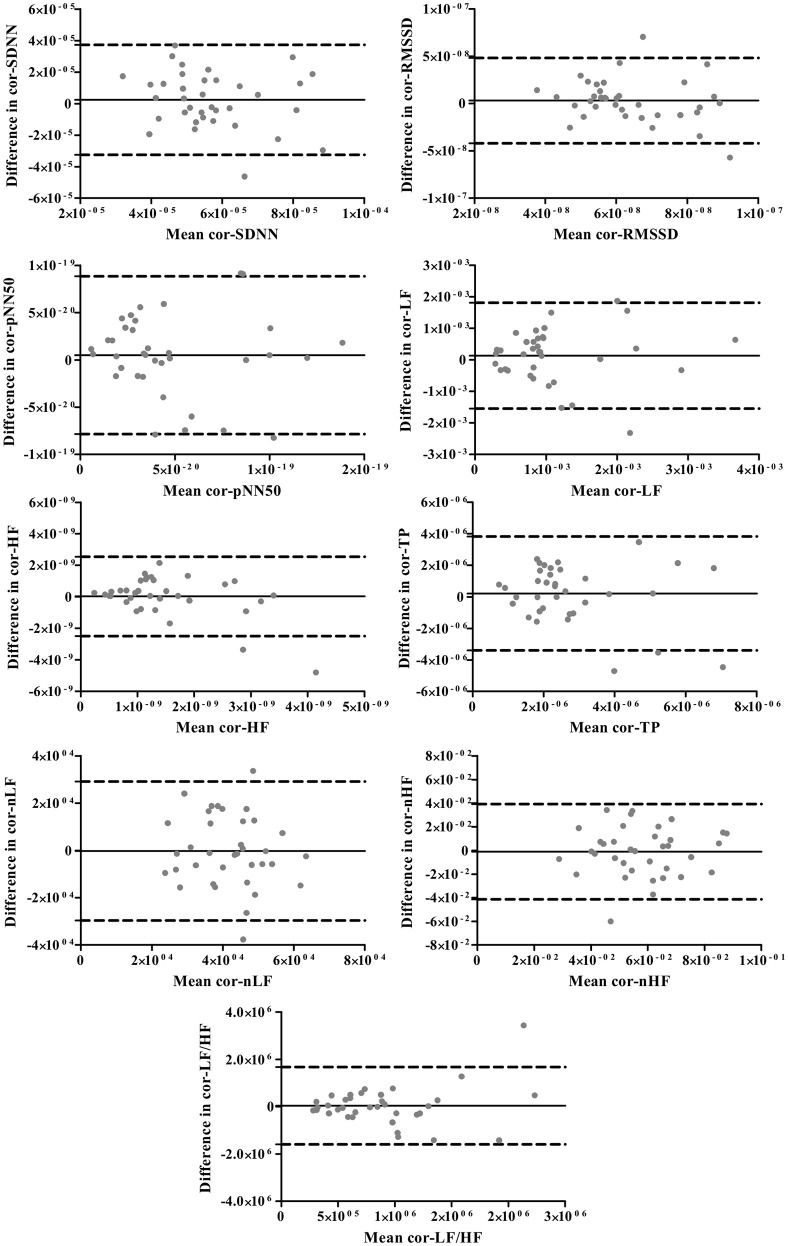
**The Bland-Altman plots for corrected HRV parameters**. The solid lines indicate the bias, and dotted lines are the 95% limits of agreement (±1.96 *SD*).

The Bland-Altman plots for standard and corrected HRV parameters are exhibited in Figures 1, 2, respectively. The differences in HRV parameters revealed a nearly symmetrical distribution around the zero line indicating the absence of a systematic change as a function of the mean (Figures 1, 2).

**Figure 3:**
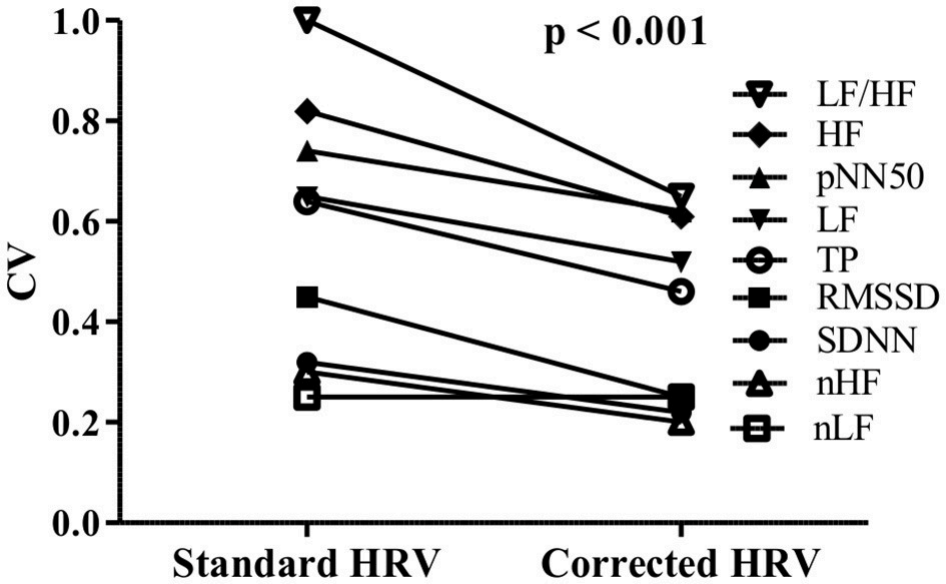
**Comparison between coefficients of variation (CV) of standard and corrected HRV parameters**.

The corrected HRV parameters presented significantly lower CVs than standard parameters (Figure 3). Of note, after the correction procedure, the coefficients of variation of HRV parameters dropped on average by 26.8%—in other words, by the same extent the reproducibility of HRV measurements improved.

## Discussion

Direct comparison of studies investigating the reproducibility of HRV is not straightforward since they are usually very heterogeneous and differ in measurement conditions including the duration of ECG recordings, time interval between test and retest as well as methods used to assess repeatability (Sinnreich et al., 1998; Carrasco et al., 2003; Sandercock et al., 2005; McNames and Aboy, 2006; Pinna et al., 2007; Tannus et al., 2013). In our experiment the ECG recordings were repeated 1 week after the initial examination, and comparing with other healthy populations (Sandercock et al., 2005), the repeatability of the standard HRV parameters was not very high (CV between 0.3 and 0.8).

A number of factors may influence HRV and its repeatability. Some of them, such as day time, ECG recording duration, measurement conditions (e.g., temperature, humidity, subject's position) can be controlled, whereas others like stress or restlessness cannot. One of the factors which is associated with all aforementioned conditions and may considerably influence HR and HRV is respiration (Billman, 2011; Quintana and Heathers, 2014; Quintana et al., 2016). Therefore, it is critical to consider respiration changes in studies where HRV measurements are repeated over a period of time (Quintana et al., 2016). To measure breathing frequency and avoid potential influence of external tools, such as mask or belt that may alter respiration depth and frequency, a compromise solution is to estimate respiratory rate from an ECG signal (Sinnecker et al., 2014; Quintana et al., 2016).

In our study we calculated the respiration rate from ECG signals and investigated its relationship with HR and HRV, as well as its influence on the HRV reproducibility. The respiratory rate was significantly and positively associated with HR what is in agreement with other observations where a decrease in breathing frequency corresponded with a lengthening of the heart period (Bruce, 1996). Akin to HR, RespRate was negatively related with HRV, however, the association between RespRate and HRV was weaker than the association between HR and HRV. Importantly, in the multivariate analysis, the HR changes proved to be main determinants of HRV reproducibility—the differences in RespRate did not exert an independent impact on HRV changes (Table 2). Moreover, the removal of the HR impact on HRV (i.e., the correction procedure) resulted in a decrease of the association between RespRate and HRV and a reduction of the influence of RespRate-diff on HRV repeatability—i.e., without HR-diff the regression models became insignificant (Table 3). It seems to be possible that the relationship between RespRate and HRV is, to some extent, operated by HR. It is hard to conclude whether HR determines RespRate or, conversely, whether HR depends on RespRate. Indeed, the cardiorespiratory interaction has been regarded in several different ways, i.e., as primarily respiration-to-heart rate (Rosenblum et al., 2002; Zhu et al., 2013) heart rate-to-respiration (Larsen et al., 1999; Tzeng et al., 2003) or bidirectional (Porta et al., 2013)—however, these differences probably depend on the different analytical technique employed (Quintana and Heathers, 2014). Coupling between respiration and HR is an important aspect of HRV analysis and requires consideration in any HRV study, particularly that the respiratory frequency can be obtained in almost every case from ECG signals.

Yet, the most important finding in our study is that the HR differences in two ECG examinations proved to be the most important factor influencing the reproducibility of the short-term HRV measurements. It is worth noting that even a very minimal change in average HR (i.e., by 1 bpm) yielded pronounced changes in HRV (i.e., 16.5% on average). This is due to the strong HRV dependence on HR. However, the association between HRV and HR is not only a physiological phenomenon but also a mathematical one (Sacha, 2013, 2014a,b,c; Sacha et al., 2013a,b,c, 2014; Billman et al., 2015). The physiological determination stems from the autonomic nervous system activity, especially from its parasympathetic branch, i.e., the higher parasympathetic activity, the slower HR and the higher its variability (Task Force of the European Society of Cardiology and the North American Society of Pacing and Electrophysiology, 1996). The mathematical determination is caused by the non-linear (inverse) relationship between R-R intervals and HRs—consequently, the same changes of HR cause much higher fluctuations of R-R intervals for the slow average HR than for the fast one (Sacha and Pluta, 2008; Sacha, 2013, 2014a,b,c; Sacha et al., 2013a,b,c, 2014; Billman et al., 2015). Recently, several methods of the HRV correction for HR have been proposed (Sacha et al., 2013a,c; Monfredi et al., 2014; Estévez-Báez et al., 2015) and the study employing one of them has demonstrated a significant improvement in the reproducibility of corrected HRV (Sacha et al., 2013c). Moreover, other studies have shown that a complete removal of the HR impact on HRV may increase the HRV prognostic power for non-cardiac death in patients after myocardial infarction (Sacha et al., 2013b, 2014). On the other hand, one can also enhance the HR impact on HRV applying mathematical modifications—such a manipulation turned out to improve the HRV prediction ability for cardiac mortality (Pradhapan et al., 2014; Sacha et al., 2014). Hence, HR seems to be a critical player in the clinical significance of HRV. In view of these reports, it is hard to determine which kind of HRV, i.e., independent (corrected) or highly dependent on HR, is more clinically relevant. In general, it is possible that for outcomes and populations where HR is not a risk factor, the removal of the HR impact improves the HRV predictive value (Sacha, 2013, 2014a,b,c; Sacha et al., 2013b, 2014). However, if for some outcomes HR is a risk factor, the enhancement of its influence makes HRV a better predictor (Sacha, 2013, 2014a,b,c; Sacha et al., 2013b, 2014).

The present study indicates that even small alterations of HR may markedly change standard HRV. Such a strong HR influence on HRV creates some therapeutic possibilities to modify HRV, i.e., by pharmacologic or non-pharmacologic reduction of HR one may augment standard HRV. In fact, this was observed in studies employing the treatment with beta-blockers which was associated with some benefit in patients after myocardial infarction (Sandrone et al., 1994; Lurje et al., 1997; Melenovsky et al., 2005). However, it remains to be determined whether HRV really increases during chronotropic interventions or this is only a mathematical consequence of HR decrease as an effect of the non-linear relationship between R-R interval and HR (Sacha and Pluta, 2008; Sacha, 2013, 2014a,b,c; Sacha et al., 2013a,b,c, 2014; Billman et al., 2015)—studies employing the correction procedure should help to answer this pivotal question.

The correction of HRV seems to be critical if one aims to compare HRV among the same individuals over a long course of time, e.g., in children during their growth when their HR progressively slows down (Fleming et al., 2011). Very recent study indicates that the corrected HRV is decreasing with age in healthy children which is accompanied by the reduction of HR—as a net result, the standard HRV may remain constant in children at different ages (Gąsior et al., 2015). Nevertheless, further studies are necessary to explore and confirm these observations in other children and adolescent populations.

## Limitations

Some limitations of our study need to be acknowledged. The respiration rate was derived from ECG signals and despite the validation of such method, some inaccuracies may be expected comparing with direct respiration measurement (Sinnecker et al., 2014). The experiment was performed among healthy participants and hence the inferences cannot be extended to patients with pathological states. The homogeneity of the participants with respect to their age range does not allow to draw conclusions on wider age populations. Finally, the sample size of our cohort is moderate which may have some impact on statistical power of our analysis.

## Conclusion

Both respiration and HR influence HRV, nevertheless the influence of breathing rate seems to be, at least in part, HR dependent. The HRV correction for the prevailing HR decreases the correlation between respiratory rate and HRV. HR turns out to be a main determinant of HRV reproducibility. The exclusion of the overall HR influence on HRV improved the repeatability of HRV by about 27% in our study population. Further studies are needed to determine the role of HR and respiration in HRV in other more specific conditions among both healthy individuals and patients with various diseases.

## Author contributions

Conceived and designed the experiment: JG, PJ. The acquisition, analysis, or interpretation of data for the work: JG, JS, PJ, JZ, JP. Drafting the work or revising it critically for important intellectual content: JG, JS, PJ, JZ, JP. Final approval of the version to be published: JG, JS, PJ, JZ, JP. Agreement to be accountable for all aspects of the work in ensuring that questions related to the accuracy or integrity of any part of the work are appropriately investigated and resolved: JG, JS, PJ, JZ, JP.

## Funding

The authors and clinical organizations with which the authors are affiliated or associated did not receive any grants or outside funding in support of the research for or preparation of the manuscript, did not receive payments or other benefits from a commercial entity, do not have any professional relationships with companies or manufacturers who will benefit from the results of the present study. The aforementioned disclosure also applies to the authors' immediate families.

### Conflict of interest statement

The authors declare that the research was conducted in the absence of any commercial or financial relationships that could be construed as a potential conflict of interest. The reviewer MT and handling Editor declared their shared affiliation, and the handling Editor states that the process nevertheless met the standards of a fair and objective review.
